# Improvement *of* sugarcane for borer resistance using *Agrobacterium* mediated transformation of *cry1Ac* gene

**DOI:** 10.1080/21645698.2020.1809318

**Published:** 2020-08-30

**Authors:** Eldessoky S. Dessoky, Roba M. Ismail, Nagwa I. Elarabi, Abdelhadi A. Abdelhadi, Naglaa A. Abdallah

**Affiliations:** a Department of Biology, Faculty of Science, Taif University, Taif, Kingdom of Saudi Arabia; b Plant Genetic Transformation Department, Agricultural Genetic Engineering Research Institute (AGERI), Agricultural Research Center (ARC), Giza, Egypt; c Faculty of Agriculture, Genetics Department, Cairo University, Giza, Egypt

**Keywords:** Sugarcane, *Agrobacterium tumefaciens*, *cry1Ac*, borer-resistance, insect-resistance

## Abstract

The sugarcane (*Saccharum* X *officinarum*) is one of the most important crops used to produce sugar and raw material for biofuel in the world. One of the main causes for sucrose content and yield losses is the attack by insect. In this investigation, *cry1Ac* gene was introduced into sugarcane variety GT54-9(C9) using the *Agrobacterium tumefaciens* transformation method for transgenic sugarcane production presenting insect-resistance. The *A. tumefaciens* strain GV1303 including pART*cry1Ac* vector was used for the production of transformed sugarcane. The *Bacillus thuringiensis cry* gene were successfully used to produce transgenic plants used for the improvement of both agronomic efficiency and product quality by acquiring insect resistance. PCR and Southern hybridization techniques were used to confirm the *cry1Ac* gene incorporation into sugarcane genome. Transformation percentage was 22.2% using PCR analysis with specific primers for *cry1Ac* and *npt-II* (Neomycin phosphotransferase) genes. The expression of *cry1Ac* gene was determined using reverse transcriptase polymerase chain reaction (RT-PCR), QuickStix test, and insect bioassays. Bioassays for transformed sugarcane plants showed high level of toxicity to *Sesamia cretica* giving 100% mortality of the larvae. Sugarcane insect resistance was improved significantly by using *cry1Ac* gene transformation.

## Introduction

Sugarcane is a paramount sugar plant vastly cultivated in the subtropical and tropical regions. It supplies about eighty percent of the sugar in the world.^1^ Moreover, in many countries sugarcane is also considered a main raw material for the production of ethanol.^2,3^ Cultivated sugarcane varieties are hybrids from the cross between *Saccharum spontaneium* (2 n = 36–128) and *Saccharum officinarum* (2 n = 20–122) which represent complex aneupolyploid.^4^ It is an octaploid species have complex genome (x = 10 and 2 n = 80 ~ 270).^5^ Insect pests are an essential problem for sugarcane crop all over the world. One of the significant pests of sugarcane is lepdoptera stem borers.^6^ The major Lepidopteran insect pests of sugarcane are stem borer (*Diatraea saccharalis*) in South America, central America, the Caribbean, and the southern United States,^7^ root borer (*Emmalocera depressalis*) in India and Pakistan, sugarcane top borer (*Chilo terrenellus*) in Bangladesh, Thailand and Australia,^8^ pink borer (*Sesamia inferens*) in ASIA, Cambodia, China, Hong Kong, India, Mexican rice borer (*Eoreuma loftini*), and pink stem borer *Sesamia cretica* in Mediterranean basin and extends through the Middle East and Arabia to Pakistan, northern India, and northern Africa extending south to northern Kenya and northern Cameroon. These borers cause yield losses of nearly 25–30%.^9^

To improve economic traits in agriculture many traditional plant breeding techniques can been used but these techniques can be time consuming, especially for genomic complex crop such as sugarcane. Moreover, conventional breeding to develop insect-resistance in sugarcane is limited by the lack of resistance available in the crop germplasm. One of the effective and economic strategies for improvement the resistance of different plants to insects is introduction of insect-resistant genes including *Bt* genes.^10^ Insect resistance could be improve by using genetic engineering approaches^11,12^ and could help in the development of sugarcane varieties production. *Bacillus thuringiensis* (*Bt*), is a Gram positive and spore-forming bacteria. During sporulation, it produce a crystalline parasporal body that shows biocidal activity against some invertebrate orders at larval stage including dipteran, *lepidopteran* and *coleopteran* insects.^13^ It was first discovered in 1901 by Gill.^14^ There are many reports that successfully obtained insect resistant transgenic sugarcane lines through introduced *Bt* genes.^15^ A lot of other crop species has been developed through *cryA*(b) gene introducing; those plants such as rice,^16^ cotton,^17^ tomato,^18^ potato,^19^ corn,^20^ and sugarcane.^21^

The first research with the aim of introducing *Bt* gene into sugarcane to produce insect resistance plants used the *cry1Ab* gene.^21^ Lately, *cry1Ac* gene was introduced into sugarcane genome by Gao^15^ that successfully obtained insect- resistant transgenic events. Wang^22^ introduced both the *EPSPS* and *cry1Ab* genes into sugarcane genome and obtained transgenic lines with herbicide tolerance and insect resistance. Recently, many monocotyledonous species used *Agrobacterium* transformation method including rice, maize, wheat, and barley.^23^ The transformation by *Agrobacterium* have diverse advantages, including minimal DNA rearrangement in transformants, technical simplicity and the capability to transfer long fragment of DNA. Although the *Agrobacterium* method has been used also in sugarcane,^24,25^ the shortage of a reproducible result has been an obstacle to found effective transformation method for routine genetic manipulation in the crop. The present study aimed to improve the borer resistance in sugarcane plants *via* introducing the *cry1Ac* gene through *Agrobacterium* transformation.

## Results

**Figure 1.**
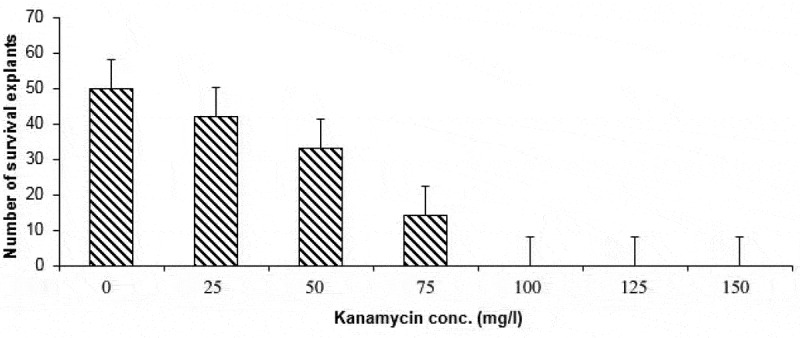
The effect of different kanamycin on the survival explants

In order to use kanamycin as a selectable marker gene in plant transformation, suitable concentration of kanamycin that inhibits explants growth should be determined.^26^ Young leaves segments were placed into media containing different concentrations of kanamycin ranging from 25 up to 150 mg/l, whilst the control was placed on kanamycin free medium. The results indicated that the number of survival explants decreased with the increasing of the kanamycin concentration. The lethal kanamycin dose for the leaf segments was found to be 100 mg/L (Figure 1).

## *Agrobacterium* Transformation

**Figure 2.**
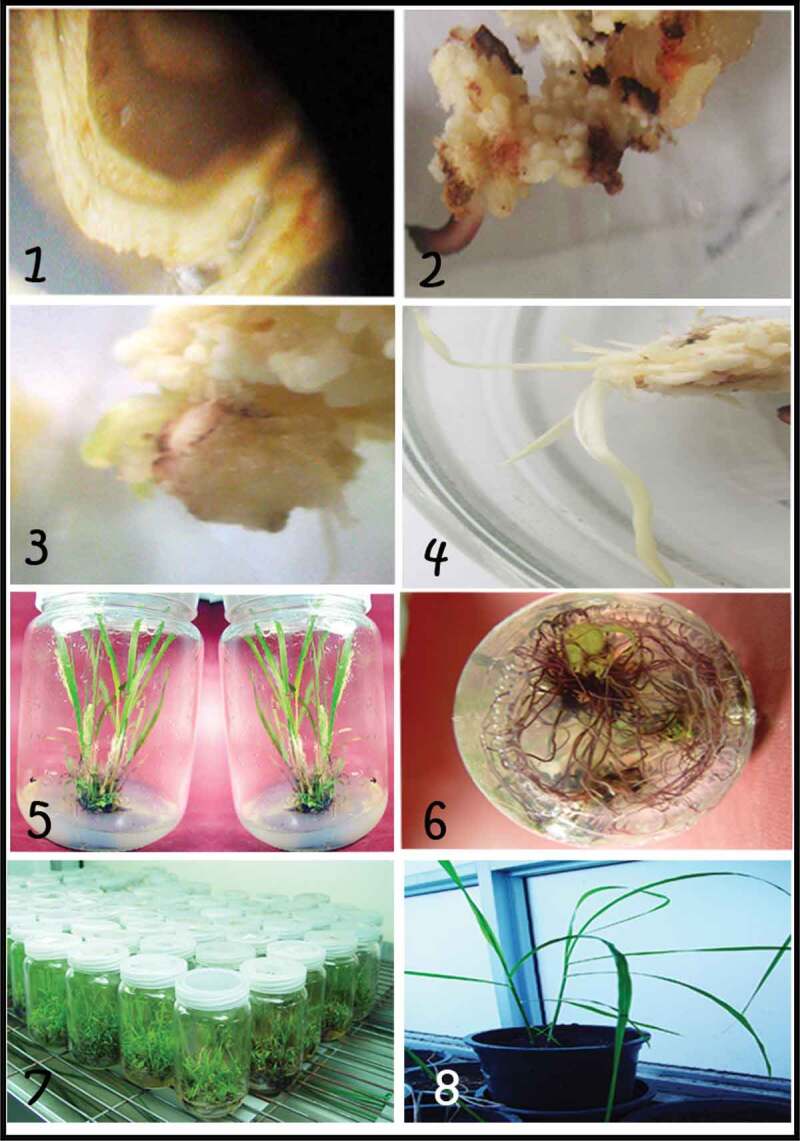
The regeneration stages of transformed sugarcane plants via direct organogenesis. (1) Leaf young explant taken from 6- to 8-month-old sugarcane (2, 3, and 4) stages of young leaf explant. (5) Shoots formation. (6) Root formation. (7 and 8) Acclimatization of transformed sugarcane plants

The sugarcane variety GT54-C9 is very popular among Egyptian farmers due to its high yield and other desirable agronomic.^27^ Sugarcane variety GT54-9(C9) were used for *Agrobacterium* transformation. Young leaf explants of sugarcane variety were co-cultivated with *Agrobacterium tumefaciens* GV3101 harboring the binary vector pART*cry1Ac*. After three days of co-cultivation, the inoculated explants were transferred to MS regeneration medium including 1 mg/l BAP and 2 mg/l NAA. After 48 hours in the dark at 28 ± 2°C, a sterile solution of strength MS medium with 300 mg/L carbenicillin was used to wash the explants then blotted briefly on sterile filter paper in the laminar flow hood. Explants were transferred to regeneration media supplemented with 100 mg/L kanamycin and 300 mg/L carbinicillin. The cultures were incubated under the regeneration conditions. After 30 days of incubation, shoots were subcultured to fresh regeneration medium with the same antibiotics in the selection plates, and were reincubated under the same conditions. Young leaves from nontransformed sugarcane were used as control. For inducing roots, regenerated shoots (about 7–10 cm) were transferred to MS medium supplemented with 60 g sucrose and 2 mg/l NAA. Regenerated plants with well-developed roots were transferred to pots containing sand, peat-moss and clay (1:1:1) and kept in a greenhouse under shadow for 15–20 days for acclimatization (Figure 2). For further analyzes, sixteen regenerated transgenic sugarcane lines were used. From a single shoot bud, a transgenic line was develop and grew on MS media containing kanamycin. Based on the morphological parameters and molecular evaluation using ISSR marker, no phenotypic abnormalities appeared in the putative transgenic plants in comparison to the untransformed control plants (data not shown).

## Detection of Transgenic Sugarcane

The transformed kanamycin resistant sugarcane shoots were used for DNA isolation. The polymerase chain reaction (PCR) was used to confirm the *cry1Ac* gene integration into the genetic material of the putative kanamycin resistant shoots (transgenic) of sugarcane cultivar GT54-9(C9) using *nptII* and *cry1Ac* specific primers. The selected primers were designed to amplify fragments of 250 and 497 bp of the *nptII and cry1Ac* genes, respectively. Out of 90 plants examined from kanamycin resistant tissues only 20 gave positive results (The PCR test showed a clear band corresponding to the relevant sequence of both primers) with a percentage of 22.2%.

**Figure 3.**
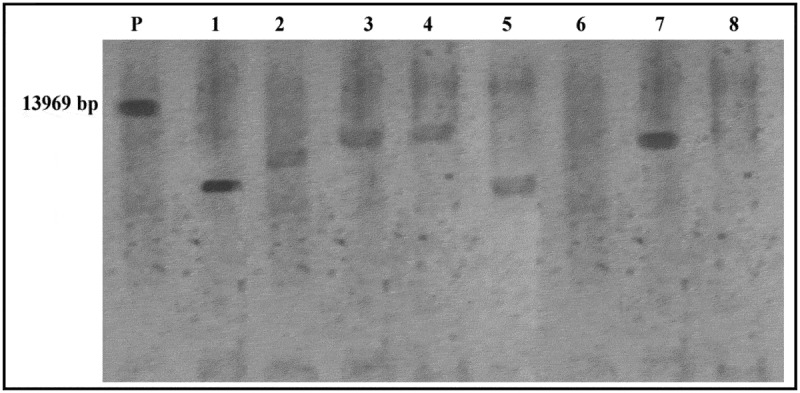
Southern blot hybridization of genomic DNA isolated from transgenic sugarcane plants, transformed by the *cry1Ac* gene. P; the liner pART*cryAc* plasmid (positive control) and lanes 1–8 are the *cry1Ac* transgenic sugarcane plants

Southern blot is a commonly used technique to confirm gene integration and copy numbers in transgenic plants. Southern blotting analysis was used to confirm the integration of the *cry1Ac* gene in the T1 sugarcane plants. A restriction enzyme, *Kpn*I was used to digest sugarcane genome and then digested DNA was hybridized with cry1Ac specific probe that showed the integration of *cry1Ac* gene in sugarcane genetic material. Liner pART*cry1Ac* plasmid DNA was used as positive control. Southern blotting test for transgenic plants showed bands >3256 bp molecular weight as expected (Figure 3).

## Expression of *cry1Ac* Gene in Transgenic Sugarcane Plants

**Figure 4.**
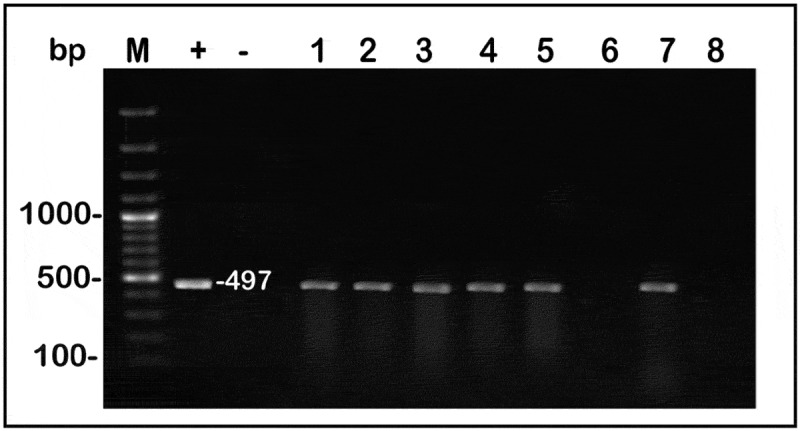
RT- PCR isolation of the *cry1Ac* gene of transformed sugarcane. M: 100 bp DNA ladder marker. Lanes (1, 2, 3, 4, 5, and 7) represent putative transformed Sugarcane plants, Lane (-) (nontransformed sugarcane) negative control, Lane (+) (pARTcry1Ac vector) positive control

The stable expression of the *cry1Ac* gene in the transgenic sugarcane lines was confirmed by using reverse transcription-polymerase chain reaction (RT-PCR). Total RNA was extract from PCR-positive putative transgenic lines and also from the nontransgenic plants (as negative control). Extracted RNA was used as a template in RT-PCR for synthesizing the cDNA followed by amplification of the *cry1Ac* gene with *cry1Ac* specific primer (Table 1). The results showed that a RT-PCR fragment with a molecular size of about 497 bp was amplified from total RNA isolated from transformed plants (Figure 4). The RT-PCR analysis for the sugarcane plants showed the occurrence of the mRNA for the *cry1Ac* gene in 6 out of 8 (75%) PCR-positive plants for sugarcane.

**Table 1.** The primer sequences of transgenes used for confirmation of T-DNA integration in putative transformed plantlets and RT-PCR

Genes	Sequences 5`- 3`	Expected Size
*npt-II*	F- CGCAGGTTCTCCGGCCGCTTGGG TGG	250 bp
	R- GACTTCGCCTTCCCTGACCGACGA	
*cryA1c*	F- GCATCTTCGGCCCGTCCCAGTC	497 bp
	R- ACGCGCTCCAGGCCGGTGTTGTA

## Survey with Trip Tests for the Cry1Ac Protein

**Figure 5.**
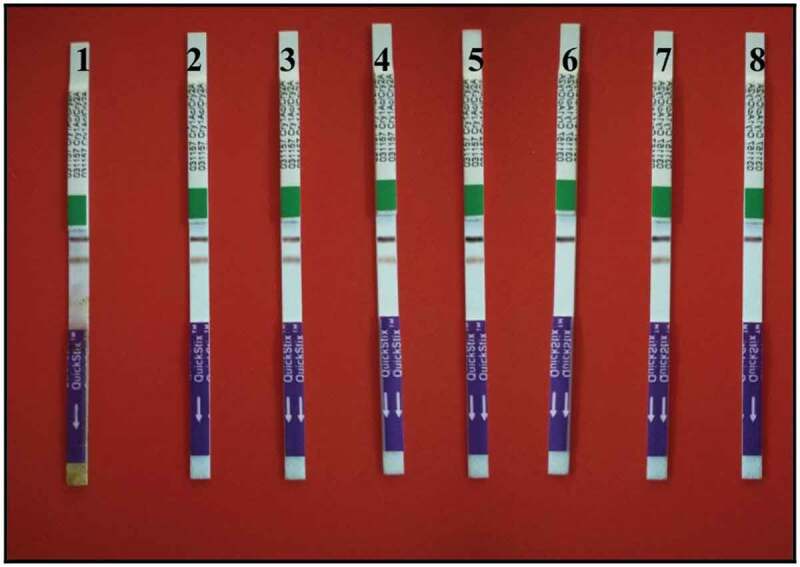
Cry1Ac protein expression in transgenic sugarcane on QuickStix Combo strips. Lines 6 and 8 showed negative results (one band). Lines 1, 2, 3, 4, 5, and 7 showed positive results (two bands)

The QuickStix test was done with Cry1Ac to detect the expression of the Cry1Ac protein in eight transgenic sugarcane lines. In the strip containing lines 6 and 8, only the assay band was observed. This indicated the absence of Cry proteins and presence of analyze. It also indicates that two spurious bands are not formed due to analyze. In samples containing the cry1Ac (lines 1, 2, 3, 4, 5, and 7) both assay band and expression band were detected as shown in Figure (5). This indicated that those lines containing the Cry1Ac protein.

## Bioassay

**Figure 6.**
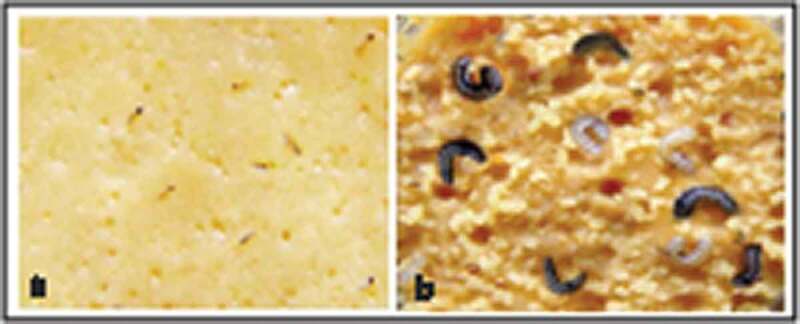
Determine the mortality percentage of Cry1Ac toxin protein against *Sesamia cretica*. (a) Mortality reached 100% at 1000 ppm of dried transformed sugarcane leaves compared to the (b) control

It is important to evaluate potential insect-resistant transgenic plants for insect resistance against target pest(s) at field conditions and conducting insect laboratory bioassays. Figure (6) shows the mortality percentage of *Sesamia cretica* caused by transgenic plants expressing cry1Ac toxin. The results indicated that the lethal concentration 50 (LC_50_) value for cry1Ac toxin protein from transformed sugarcane plants were 500 ppm (line 12, 15) and 300 ppm (line 5, 8, 14, 16) against the *Sesamia cretica* in 6 transformed plants. The mortality percentage of cry1Ac toxin expressed in all transgenic plants against *Sesamia cretica* were 100% with 1000 ppm compared to the negative control (Table 2). This data indicated the high expression of *cry1Ac* gene in the transformed sugarcane plant. The transgenic plants showed higher resistant to the target pests. However, the line 8 showed the highest toxicity to the larvae at lower concentrations followed by line 16. Therefore, these two lines are recommended for using to further experiments to control the steam borer *Sesamia cretica*.

**Table 2.** Mortality percentages of *Sesamia cretica* fed on nontransgenic (control) and six transformed sugarcane of expressing the *cry1AC* gene

	% mortality/Concentration (ppm)					
Selected sugarcane lines	1000	700	500	300	200	Lethal concentration 50 (LC50)
Nontransformed	0%	0%	0%	0%	0%	-
5	100%	80%	60%	50%	20%	300 ppm
8	100%	100%	80%	60%	40%	300 ppm
12	100%	70%	50%	40%	20%	500 ppm
14	100%	70%	60%	50%	30%	300 ppm
15	100%	80%	60%	40%	30%	500 ppm
16	100%	90%	70%	50%	20%	300 ppm

## Discussion

Transgenic plants expressing *cry* genes from *Bacillus thuringiensis* could drastically minimize the use of broad-spectrum insecticides against insect pests. Endotoxins produced by *Bt* strains have insecticide effect for some of the main pests of important crop plants.^28^ Genetically modified plants expressing *Bt* genes are more effective in controlling to pests than *Bt* formulations. In 1995, the Environmental Protection Agency (EPA) in the United States approved the commercial production and distribution of the *Bt* crops: corn, cotton, potato, and tobacco.^29^ Recently, *Bt* soybean varieties expressing the *cry1Ac* gene has been approved for commercial use in Latin America^30^ and *Bt* sugarcane CTC175 has been approved for commercial use in Brazil.^31^ The use of synthetic insecticides was significantly reduced by using commercialization *Bt* crops.^32^ A lot numbers of *cry* genes have been tested and described against main insect pests.^33^ Many research suggested that the toxicity of cry toxins was due to either their pore formation ability^34^ or the signal transduction pathway by receptor binding.^35^ The mode of action model of *Bt* genes suggest that cry toxins pass through a successive binding mechanism with several insect gut proteins leading to membrane insertion, pore formation, and toxin oligomerization.^35^ The cry toxin produced as protoxin in *Bt* bacterial cells. A high yield of an active three-domain toxin of *Bt* cry toxins can be produced by insect gut proteases enzymes. Both *cry1A* protoxin and activated toxin binds to cadherin receptor forming distinct oligomers that insert into the membrane forming lytic pores. The outcome of this double mode of action is the decreasing of possibility evolution of resistance and possibly to expand the spectrum of insect targets. For the continues use of *Bt* crops, it is probable that obtaining a stable expression of cry full length proteins will have the same outcome, delaying resistance and protection from a broad number of insect pests.^36^ The first reported of the *cry1Ac* gene expression using *Agrobacterium* transformation method was in cotton for insect resistant to bollworm (*Helicoverpa armigera*).^37^ In sugarcane, *Agrobacterium* mediated genetic transformation is considered to be reliable method and more efficient than direct biolistic gene transfer method.^38^ *Agrobacterium* mediated genetic transformation has traditionally been the preferred method to generate events with low transgene copy number. Standard biolistic gene transfer method, in which large quantities of whole plasmid constructs are introduced, typically result in the integration of multiple transgene copies as well as vector backbone sequences into the plant genome.^39^ In this investigation, sugarcane variety GT54-9(C9) was transformed with *A. tumefaciens* GV3101 that have pART*cry1Ac* binary vector. The target gene integration and expression in the plant genomic DNA are important reasons for success of transformation as well as its inheritance in progeny plants. The stable integration of the *cry1Ac* gene into sugarcane DNA was assured by using the PCR and Southern blot analysis. The results showed that only twenty plants from ninety gave specific bands (250, 497 bp) corresponding to both *nptII* and *cry1Ac* specific primers, repressively about 22.2% were transformed sugarcane plants. The copy number determination of transgenes in transgenic plants is important due to the effect of the copy number on the gene expression level and genetic stability. Southern blot analysis is considered the traditional method to assess copy number of exogenous genes in transgenic plants. In this study Southern results showed the integration of *cry1Ac* gene in the sugarcane genetic material. However, the gene copy number was integrated in one to three position in sugarcane plants that were transformed using *Agrobacterium* method.^24,40,41^ The cry1Ac toxin demonstrated to be expressed by transgenic sugarcane plant and it remains biologically active when absorb by the target insects. Quickstix was used to quantification determination of cry proteins. RT-PCR analysis was used to confirm the expression of the *cry1Ac* gene. RT-PCR technique can be used to determine the presence or absence of specific transcript and the steady-state RNA levels. Falco and Silva-Filho^42^ used RT-PCR in transformed sugarcane plants to reveal the expression of *cry1Ac* gene and detected that all plants expressed mRNA of the transgene.

Clear effects of *cry1Ac* expression were tested by the death-rate of *Sesamia cretica* when it was fed on transgenic *Bt* sugarcane. These results showed that a large amount of *Bt* protein was found in all sugarcane transgenic lines and that for target lepidopteran insect pests management the plants expressing *cry1Ac* gene could be used. The mortality percentage of *cry1Ac* toxin expressed in all transgenic plants against *Sesamia cretica* were 100% with 1000 ppm compared to the negative control. This data indicated the high expression of *cry1Ac* gene in the transformed sugarcane plant. The results indicated that sugarcane line 8 showed the highest toxicity to the larvae at lower concentrations followed by line 16 this may be due to the integration of only one cope number of the gene due to the result of Southern blot analysis. Many earlier researchers found that the multiple T-DNA insertions had exhibited less expression levels than single copy transgenes.^43^ These results also are similar to Lin *et al*,^43^ they found that bioassays with *cry1A*c transformed transgenic tobacco plants showed high level of toxicity toward (*Spodoptera litura*) giving rate of 76.9 to 100% mortality of the larvae after 72 hr. Earlier, sugarcane cultivars, CoJ 64 and Co 86032 was modified by the *cry1Ab* gene.^44^ The percentage of *cry1Ab* protein in several transgenic lines ranged from 0.007% to 1.73% for total soluble protein in leaves. At the seedling stage, transgenic plants had significantly less dead rate and there was a negative relation between the dead rate and protein expression. Weng *et al*.^45^ found that only resistance was showed in transgenic sugarcane lines expressing the *cry1Ac* protein more than 9 ng/mg when they analyzed pest resistance of *cry1Ac*. A positive correlation between pest resistance and the *cry1Ac* content was also detected.

## Conclusion

The transgenic sugarcane with *cry1Ac* gene that has been inserted into sugarcane genetic material by *Agrobacterium* transformation method, showed resistant to insects and high productivity. From different molecular analyzes confirmed that crystal protein gene is stable integrated into transgenic sugarcane genome.

## Materials and Methods

### Plant Material

Sugarcane variety GT54-9(C9) was obtained from Sugar Crops Research Institute, Agricultural Research Center, Giza, Egypt. Young leaf explants (apical part of the shoot) 3 cm in length of several layers of leaves taken from 6- to 8-month-old sugarcane (*Saccharum officinarum* cv. GT54-C9). The outer leaves were removed to expose the six inner leaves. innermost six leaves were sterilized in 70% ethanol for one min, disinfected in 40% clorox solution for 20 minutes 40% (v/v), then washed four times in sterile distilled water followed by removing the outer 6^th^ and 5^th^ leaves. Eight cm segments from the bases of the innermost three or four leaves were cut into small transverse sections (2–3 mm) and used as explants.

### Bacterial Strains and Vector

**Figure 7.**
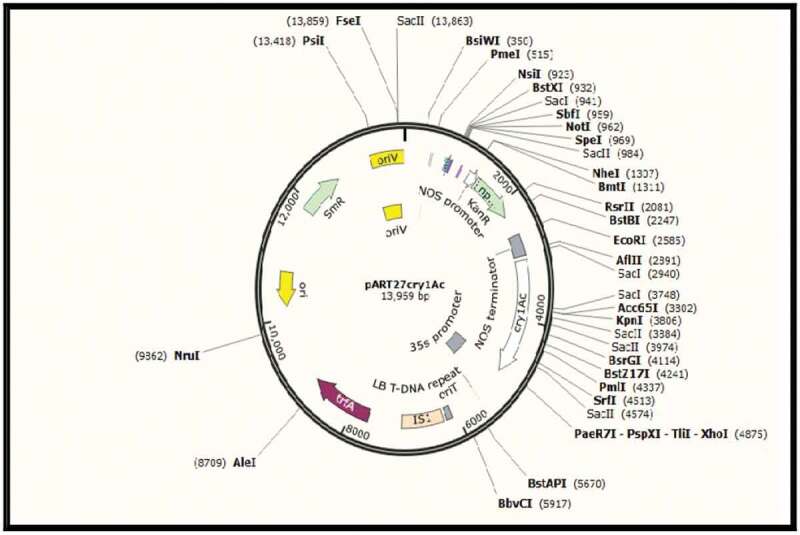
The map of pART*cry1Ac* vector

*Agrobacterium tumefaciens* GV1303 strain containing pART*cry1Ac* plasmid was used to transform sugarcane explants for produce insect resistance plants. The plasmid was constructed by Prof. Dr. Naglaa Abdallah from cloning the synthetic *cry1Ac* gene accession number AF023672.1 that was kindly provided by Dr. Pamela Green^46^ (it was modified for plants to achieve higher expression) into the pART27 binary vector and under the control of 35S promoter and was used for transforming sugarcane explants using *Agrobacterium* method (Figure 7).

## Transformation and Regeneration Conditions

Young leaf explants were soaked in *Agrobacterium* solution for fifteen min, left to dry on a sterilized filter paper, and then co-cultivated on the shooting formation media (MS including 2 mg/l NAA and 1 mg/l BAP) for three days. After co-cultivation, the explants were transferred to the same medium supplemented with 300 mg/l carbinicillin to inhibit *Agrobacterium* growth in addition to 100 mg/l kanamycin for transgenic shoots selection. Developed shoots were re-cultured on the optimized elongation medium (MS supplemented 0.1 mg/l BAP and 2 mg/l Kin) to reach suitable length. The cultures were kept in the growth chamber at 28 ± 2°C under 16 hours photoperiod of 3000 Lux provided with white cool fluorescent lamps. Then the shoots were transferred to appropriate rooting media (MS including 2 mg/l NAA) and plantlets were acclimatized in the greenhouse.^47^

## Survival Curve of Kanamycin

To set the minimal lethal dose of kanamycin, different concentrations 0, 25, 50, 75, 100, 125, 150 mg/l kanamycin was added to MS medium and 90 explants were used for each concentration. Kanamycin was sterilized using disposable filters (0.22 µn) and mixed with precooled (45–50°C) autoclaved MS medium. The percentages of explants survival (kanamycin resistant) were recorded after 21 days from culturing.

## PCR Conformation

The genetic material of the putative transgenic tissues were extracted *via* CTAB method according to Lassner.^48^ Two specific oligonucleotides primers for *cry1Ac* and *npt-II* genes were used to confirm the stable integration of the T-DNA into sugarcane genome in PCR reaction (Table 1). The DNA Synthesizer 392, Applied Biosystems at AGERI, ARC, Giza, Egypt was used for primers manufacturing. The PCR reaction was prepared in a 50 µl consisting of a final concentration of each of the following: 200 µM of each of dNTPs (dGTP, dCTP, dATP, and dTTP), 1 pmoles from each of the used primers, 1X PCR buffer, 0.04 U *Taq* DNA polymerase, 2 ng of plant DNA (as template), 2.5 mM MgCl_2_ and d.H_2_O. Amplification cycle program of the synthetic *Bt* gene was performed as following: 95°C for 5 min, followed by 35 cycles at 94°C for 60 sec, 55°C for 60 sec, 72°C for 60 sec, and a final extension at 72°C for 7 min. 1.5% agarose gel electrophoresis was used for PCR analysis by loading 15 µl of PCR product with 3 µl loading buffer

## Southern Blotting Analysis

Total DNA was isolated from transformed sugarcane as described previously by Lassner^48^ method. 10 µg of DNA was digested using restriction enzyme, separated by electrophoresis (using 1% agarose gels) and transferred to Hybond NC nylon membrane (Amersham, RPN119B, Netherlands) as described by Sambrook.^49^ Prehybridization and hybridization conditions were in strict accordance with the manufacturer’s recommendations. PCR produced from *cry1Ac* gene (497 bp) was used as a probes. Biotin Chromogenic Detection kits (#K0661 & #K0662, Ferments Life Sciences, USA) was used for hybridization and detection according to instructions provided by the manufacturer.

## Reverse Transcription PCR (RT-PCR) Reaction

Total RNA was extracted from PCR positive plants using SV Total RNA Isolation System (Promega, cat. #Z3100, USA). RT-PCR analysis was performed using Robust Ι RT-PCR kit, Finnzymes, Finland. The analysis were carried out on both putative transformed (PCR positive) and nontransformed plants using the *cry1Ac* gene specific primers (Table 1) and the PCR products were separated in 1.5% agarose gels.

## Survey with Trip Tests for the Cry1Ac Protein

QuickStix Combo Kit for Cry1A and Cry2A (cat. #AS 012 LS, EnviroLogix, Portland, Oregon, USA) was used to detect the presence of cry1Ac protein in transgenic sugarcane leaves. The samples and protein extract were performed according the manufacturer’s instructions.

Two leaf punches were taken from each plant by snapping cap of eppendorf tube and were grounded by rotating pestle until fine grinding. Extraction buffer (0.5 ml) was added to the tube and mixed with the leaf tissue. QuickStix strips were dipped in the leaf and examined after 10 min for the appearance of the final bands on strip and results were recorded.

## Bioassay

*Sesamia cretica* larvae’s obtained from a laboratory culture were reared on an artificial medium diet as method described by Dulmage.^50^ Transformed sugarcane plants with positive PCR results for *nptII* and *cry1Ac* were subjected for bioassay test. One gram of dried grounded sugarcane leaves was suspended in 100 ml water to give 1000 ppm and further diluted to prepare different concentrations of 1000, 700, 500, 300, and 200 ppm. A volume of 500 µl of each dilution was added onto the surface of each cup containing artificial media and three replicates from each dilution were prepared. After the toxin was completely dried and the surface become dry, 10 neonate larvae were placed on the media surface and were monitored for 96 hrs. The cups were covered with aluminum foil and left at 26°C (± 2°C). The leaves of nontransformed sugarcane plant with 500 µl water were used as negative control. The mortality was recorded every 24 hours for four days.

## Statistical Analysis

For statistical analysis, ANOVA program was used for variance analysis of data. For all treatments, significance at 5% level was used to test differences among means by using Duncan^51^ new multiple range test as described by Snedecor and Cochran.^52^ Means followed by the same letter are not significantly different at *p* ≤ 0.05.
